# CD163 and the FVIII/FXII ratio identified as novel biomarkers for early sepsis recognition

**DOI:** 10.3389/fmed.2026.1682209

**Published:** 2026-02-04

**Authors:** Nan Yan, Yanjun Diao, Shan Zhou, Linhe Lu, Chu Chen, Bingbing Zhu, Jingyuan Jia, Jiayun Liu

**Affiliations:** 1 Department of Laboratory Medicine, Xijing Hospital, Air Force Medical University, Xi’an, Shaanxi, China; 2 Department of Cardiovascular Surgery, Xijing Hospital, Air Force Medical University, Xi’an, Shaanxi, China; 3 Department of Laboratory Medicine, Shaanxi Provincial Hospital of Traditional Chinese Medicine, Xi’an, Shaanxi, China

**Keywords:** bioinformatics, CD163, coagulation, FVIII/FXII ratio, sepsis

## Abstract

**Background:**

Sepsis is a systemic inflammatory syndrome caused by infection with certain pathogens. It includes sepsis, severe sepsis, and septic shock. In the intensive care unit (ICU), sepsis has a higher mortality rate. Therefore, early identification and management of sepsis can improve outcomes.

**Objective:**

All the studies aimed to find out novel markers for early clinical recognition of sepsis.

**Methods:**

We retrospectively analyzed 1,245 results of the detection of intrinsic coagulation factors (FVIII, FIX, FXI, and FXII). All the analysis results were visualized by the boxplot, Venn diagram, and circular barplot. After downloading three datasets from the Gene Expression Omnibus (GEO), differentially expressed genes (DEGs), common genes of the three datasets, Gene Ontology (GO), and Kyoto Encyclopedia of Genes and Genomes (KEGG) pathways enrichment analysis were analyzed and shown. Next, we screened hub genes. Finally, three receiver operating characteristic (ROC) curves were plotted separately for patients of three diseases versus 20 healthy normal subjects.

**Results:**

Our analysis of 1,245 clinical specimens revealed that the co-occurrence of elevated FVIII (>150%) and reduced FXII (<50%) was most prevalent in three diseases: cirrhosis (*n* = 8), pneumonia (*n* = 5), and sepsis (*n* = 4). Multi-dataset screening identified 70 common differentially expressed genes. Protein–protein interaction network analysis pinpointed CD163 as the top hub gene (degree = 13). Critically, ROC analyses demonstrated that the FVIII/FXII ratio exhibited superior diagnostic performance for sepsis (AUC = 0.920), outperforming both FVIII (AUC = 0.710) and FXII (AUC = 0.840) alone.

**Conclusion:**

CD163 and the FVIII/FXII ratio can be used as novel markers for early clinical recognition of sepsis.

## 1 Introduction

Sepsis is defined as a dysregulated host response to infection, leading to life-threatening organ dysfunction (Sepsis-3), and represents a major cause of mortality in intensive care units (1, 2). Early diagnosis is critical for improving outcomes, yet remains challenging due to the syndrome’s highly variable and non-specific early manifestations (3, 4).

This diagnostic challenge is rooted in sepsis pathophysiology, which is characterized by a maladaptive interplay between systemic inflammation and coagulation, often termed “immunothrombosis” (5–7). For instance, components of the intrinsic coagulation pathway are not only effectors of thrombosis but also active participants in inflammation: elevated FVIII is an established risk factor for thrombosis (8), while FXII activation contributes to leukocyte-mediated proinflammatory responses (9). This coagulopathy–inflammation axis is a common feature across severe inflammatory states. It underlies the frequent progression of severe pneumonia to septic shock (7, 10) and explains the exceptional vulnerability and high mortality of patients with decompensated cirrhosis following bacterial infection (11).

Given this intertwined pathology, biomarkers that simultaneously reflect both coagulation and inflammatory disturbances may offer superior diagnostic precision for early sepsis. However, the combined utility of specific markers from these pathways is underexplored. We hypothesize that the FVIII/FXII ratio—capturing a prothrombotic versus proinflammatory imbalance within the intrinsic pathway—and CD163—a soluble marker of macrophage activation reflecting immune dysregulation—together provide a more comprehensive profile of the septic “immunothrombotic” state than either marker alone.

Therefore, this study aimed to: (1) identify a distinct coagulation phenotype (FVIIIhigh/FXIIlow) through retrospective clinical screening; (2) discover key immune-related hub genes like CD163 via bioinformatics analysis of public sepsis datasets; and (3) independently validate the collaborative diagnostic value of the FVIII/FXII ratio and CD163 for the early clinical recognition of sepsis.

## 2 Materials and methods

### 2.1 Specimen selection

This study comprised two distinct cohorts for different phases of analysis: an exploratory cohort and a validation cohort.

#### 2.1.1 Exploratory cohort (phenotype discovery)

A total of 1,245 results of intrinsic coagulation factors’ (FVIII, FIX, FXI, and FXII) activity detection at the first Affiliated Hospital of Air Force Military Medical University, from January 2023 to December 2024, were reviewed for the study. From this pool, we identified all samples exhibiting the concurrent coagulation phenotype of elevated FVIII (>150%) and reduced FXII (<50%). Subsequent reviews of medical records classified these into disease categories. The exploratory cohort analysis revealed the distribution of the FVIII high/FXII low phenotype across distinct disease entities.

#### 2.1.2 Validation cohort (biomarker performance)

A dedicated validation cohort was retrospectively constituted to comprehensively evaluate the diagnostic and discriminatory performance of the FVIII/FXII ratio. To ensure complete independence from the exploratory cohort used for phenotype discovery, subjects for this validation cohort were selected from a subsequent, non-overlapping time period: January to June 2025. This cohort comprised four distinct groups:

The Sepsis Group (*n* = 20): Patients identified from hospital records who were diagnosed with sepsis according to the Sepsis-3 criteria during the validation period.

The Pneumonia Group (*n* = 20): Patients with a primary diagnosis of pneumonia identified during the same period, confirmed by radiographic and clinical criteria, without meeting sepsis criteria at sampling.

The Venous Thromboembolism (VTE) Group (*n* = 20): Patients with objectively confirmed VTE identified during the validation period, in the absence of active infection.

The Healthy Control Group (*n* = 20): Age- and sex-matched individuals with no history of active infection or coagulopathy, whose data were obtained from routine health screening examinations conducted during the same timeframe (January to June 2025).

For all patient groups, to minimize potential confounding effects on coagulation parameters, individuals with a history of anticoagulant medication use or abnormal hepatic/renal function (excluding cirrhosis) were strictly excluded. Additional exclusion criteria encompassed active malignancies, severe complications, major recent trauma/surgery/stroke, pregnancy, lactation, other targeted diseases, and comorbidities, such as hypertension and diabetes.

### 2.2 One-stage assays

CaCl₂ (Siemens) and the activated partial thromboplastin time (aPTT) reagent, Actin FSL (Siemens), were used in intrinsic coagulation factors activity (Siemens) assays. The coagulation reaction was measured using a Sysmex CS5100 automatic blood coagulation analyzer (Sysmex).

### 2.3 Screening of coagulation factors

1,245 samples were reviewed, and the number of samples with intrinsic coagulation factors activity FVIII > 150% (high) and FXII < 50% (low) was found, respectively. The distribution of the number of each intrinsic coagulation factor is shown in a boxplot.

### 2.4 Disease classification

Cross-type diseases were screened from FVIII > 150% and FXII < 50% activity detection ranges by a Venn diagram. Venn diagram was generated using the Xiantao academic tools.^1^ Circular barplot showed the number of different kinds disease and was visualized using Hiplot (12).^2^

### 2.5 Identification of DEGs for three disease datasets

The publicly available Gene Expression Omnibus (GEO)^3^ datasets for cirrhosis (GSE54238), pneumonia (GSE171110), and sepsis (GSE137342) were retrieved for integrated analysis. To address potential technical variations arising from different microarray platforms, batch correction (ComBat algorithm) and normalization were performed using appropriate bioinformatic methods prior to merging the datasets. Differentially expressed genes (DEGs) were identified using the GEO2R^4^ online tool. A conventional threshold of |log₂ fold change (FC)| > 1 and adjusted *p*-value (Benjamini-Hochberg) < 0.05 was applied to ensure the selection of biologically meaningful genes and minimize the inclusion of non-specific signals in downstream functional analyses.

Volcano plots were constructed to show the DEGs. A Venn diagram revealed that these genes were common between the three datasets. Volcano plots and Venn diagrams were constructed using the Xiantao academic tools (see text footnote 1).

### 2.6 GO and KEGG enrichment analyses

Functional enrichment analysis of the 70 common differentially expressed genes (DEGs) was performed using the “GO & KEGG Enrichment Analysis” module within the Xiantao Academic online platform.^5^ The analysis followed a defined, stepwise pipeline.

#### 2.6.1 Data input

The official gene symbols of the 70 common DEGs were uploaded as the target gene list.

#### 2.6.2 Parameter configuration

Species: *Homo sapiens* was selected; Gene ID Type: “Gene Symbol” was specified; analysis types: Both “GO Enrichment” (covering Biological Process, Cellular Component, and Molecular Function) and “KEGG Pathway Enrichment” were checked; background gene set: The default background. “All annotated genes for the selected species,” was applied; statistical thresholds: The significance cutoffs were set as adjusted *p*-value (FDR) < 0.05 and minimum gene count per term = 2.

#### 2.6.3 Execution and output

The platform executed the enrichment analysis against its built-in annotation databases. Significantly enriched terms were defined as those meeting the pre-set FDR threshold of 0.05. The results, such as enrichment statistics (Gene Ratio, *p*-value, Adjusted *p*-value, Gene List) for each term, were tabulated. Visualization plots (bubble plots) for the top enriched GO terms and KEGG pathways were generated by the platform’s integrated plotting function using default graphical parameters and were exported for presentation.

### 2.7 PPI network analysis and hub genes

The protein–protein interaction (PPI) network for the 70 common differentially expressed genes (DEGs) was constructed and analyzed following a sequential pipeline.

#### 2.7.1 Network retrieval from STRING database

The official gene symbols of the 70 common DEGs were submitted as a query to the STRING database (version 12.0).^6^ The search was configured with the following specific parameters: organism: *Homo sapiens* (9,606), meaning of network edges: evidence, and a minimum required interaction score: 0.400 (medium confidence). Only interactions designated as physical subnetworks were retrieved.

#### 2.7.2 Network visualization and topological analysis

The resulting interaction data, containing nodes (proteins) and edges (predicted or known interactions), was downloaded in ‘TSV’ format and imported into Cytoscape software (version 3.10.3) for visualization and further analysis.

#### 2.7.3 Hub gene identification

Within Cytoscape, the Cyto-Hubba (13) plugin (version 0.1) was employed to identify pivotal nodes (hub genes) in the network. Hub genes were identified using the ‘Degree’ algorithm. Nodes were ranked in descending order based on their degree score, which represents the number of direct interactions a node has within the network.

The gene with the highest degree score was defined as the top hub gene for subsequent analysis.

### 2.8 Independent validation of biomarker performance

#### 2.8.1 Timing of biomarker measurement in the validation cohort

For all patients in the validation cohort, blood samples for coagulation factor (FVIII and FXII) measurement were collected at a standardized baseline time point. This was defined as the time of initial clinical diagnosis (sepsis, pneumonia, or VTE) and prior to the initiation of any systemic antibiotic or therapeutic anticoagulation. For septic patients, this corresponded to the first blood draw after fulfilling the Sepsis-3 criteria, typically within 2 h. Relative to hospital admission, sampling generally occurred within 6 h. The time of symptom onset was not used as a reference due to its subjectivity and potential recall bias.

#### 2.8.2 Receiver operating characteristic (ROC) curve analysis

The diagnostic accuracy of the FVIII/FXII ratio was evaluated by performing three separate Receiver Operating Characteristic (ROC) curve analyses, each comparing one of the three patient groups (VTE, pneumonia, and sepsis) against the Healthy Control Group. The area under the curve (AUC) was calculated for each comparison to quantify the biomarker’s discriminatory power.

## 3 Results

### 3.1 Coagulation factor screening results

**Figure 1:**
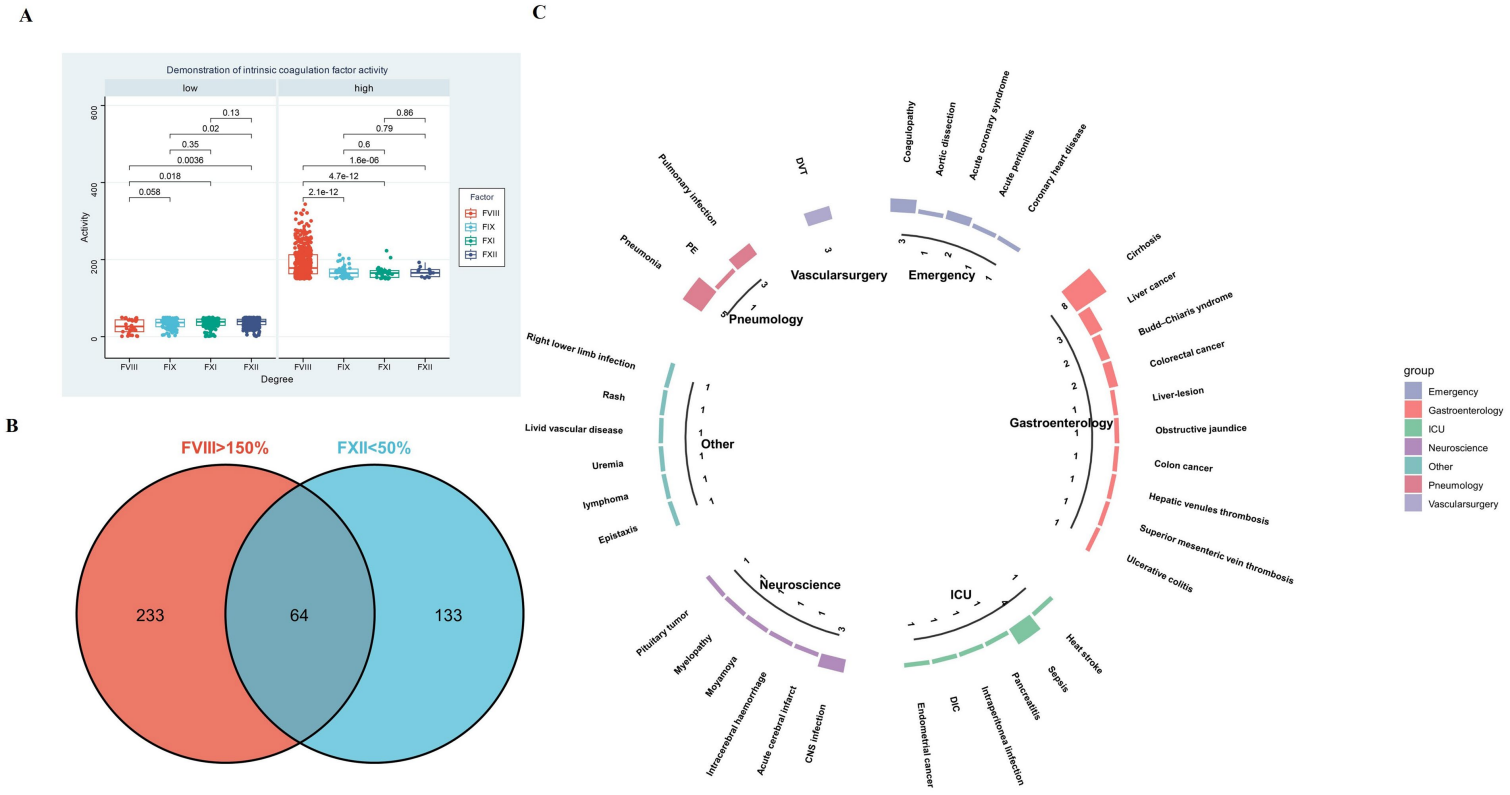
**(A)** Distribution of activity levels for intrinsic coagulation factors (FVIII, FIX, FXI, and FXII). FXII showed the highest number of samples in the low activity range (<50%; most significant *p*-value = 0.0036, *t*-test), while FVIII had the highest number in the high activity range (>150%; all *p*-values <0.0001, *t*-test). **(B)** Venn diagram displaying 64 overlapping diseases identified from samples with FVIII > 150% and FXII < 50% activity. **(C)** Circular bar plot illustrating the distribution of different diseases; the top three most frequent conditions were cirrhosis (eight cases), pneumonia (five cases), and sepsis (four cases).

It can be seen from the boxplot that the number of specimens with FVIII > 150% (high) and FXII < 50% (low) is the largest (Figure 1A). Therefore, we set out to further investigate the functional significance of FVIII and FXII.

### 3.2 Identification of the FVIII high/FXII low phenotype and its associated disease spectrum

Exploratory Cohort (Phenotype Discovery): From the initial 1,245 samples tested, we identified 64 samples that exhibited the concurrent coagulation phenotype of elevated FVIII activity (>150%) and reduced FXII activity (<50%) (Figure 1B). A subsequent review of the medical records for these 64 phenotype-positive samples classified them into clinical disease categories. A circular barplot visualizing this distribution revealed that the three most frequent diagnoses associated with this phenotype were cirrhosis (*n* = 8), pneumonia (*n* = 5), and sepsis (*n* = 4) (Figure 1C).

### 3.3 DEGs for three disease datasets

**Figure 2:**
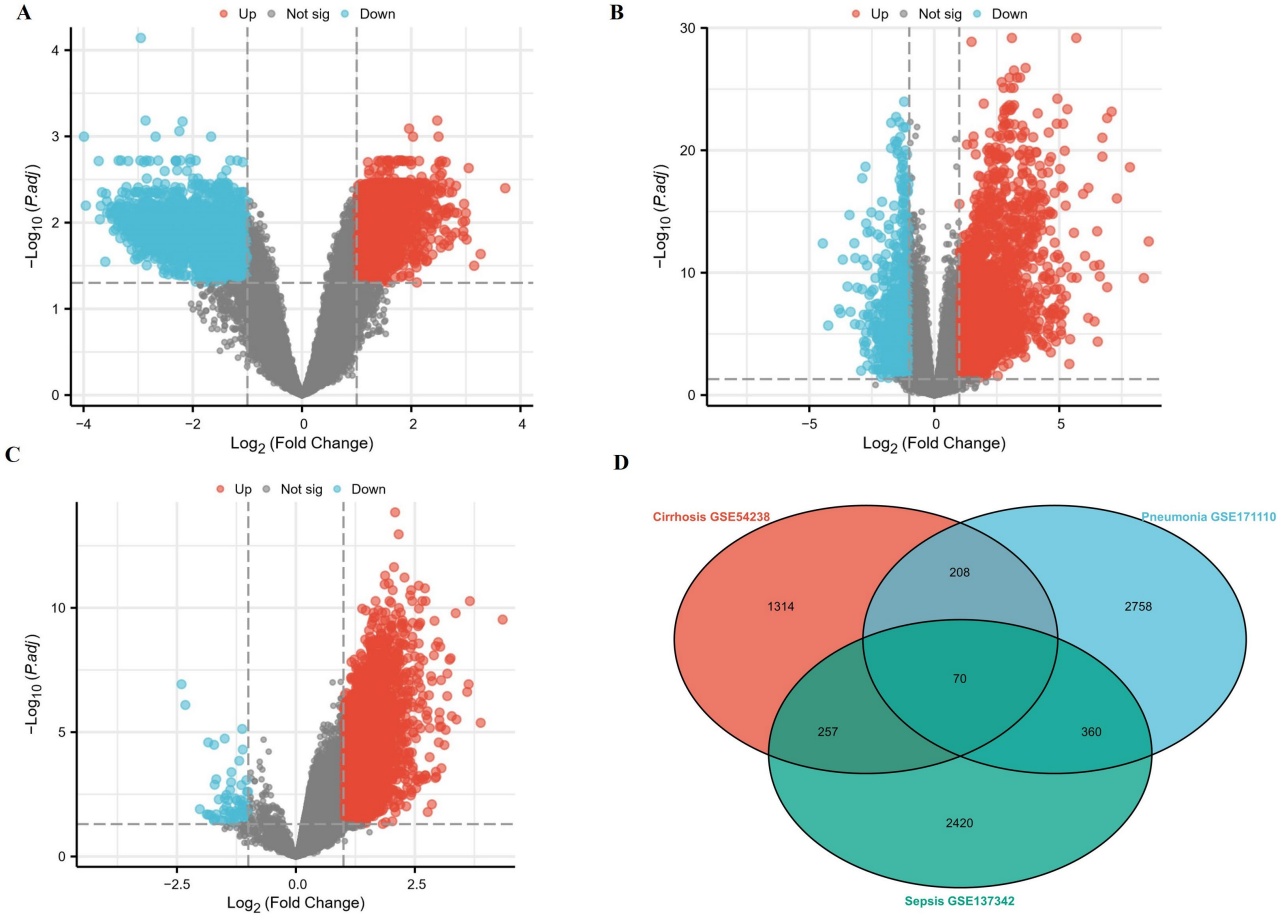
**(A–C)** Volcano plots of the differentially expressed genes from the three datasets. *X*-axis: the fold change expressed as log_2_; *Y*-axis: statistical significance (−log10(adj. pvalue)). The vertical dotted lines corresponded to twofold up and down, and the horizontal dotted line represents a *p*-value of 0.05. The red dots indicate significantly upregulated genes, and green indicates significantly downregulated genes. **(A)** Volcano plot of cirrhosis dataset (GSE54238). **(B)** Volcano plot of pneumonia dataset (GSE171110). **(C)** Volcano plot of the sepsis dataset (GSE137342). **(D)** Venn diagram of 70 common genes of all upregulated and downregulated genes in the three datasets.

From cirrhosis (GSE54238), pneumonia (GSE171110), and sepsis (GSE137342) datasets, we screened DEGs, such as upregulated and downregulated genes from three datasets, respectively. Differential expression was visualized using volcano plots (Figures 2A–C).

### 3.4 GO and KEGG enrichment analysis results

**Figure 3:**
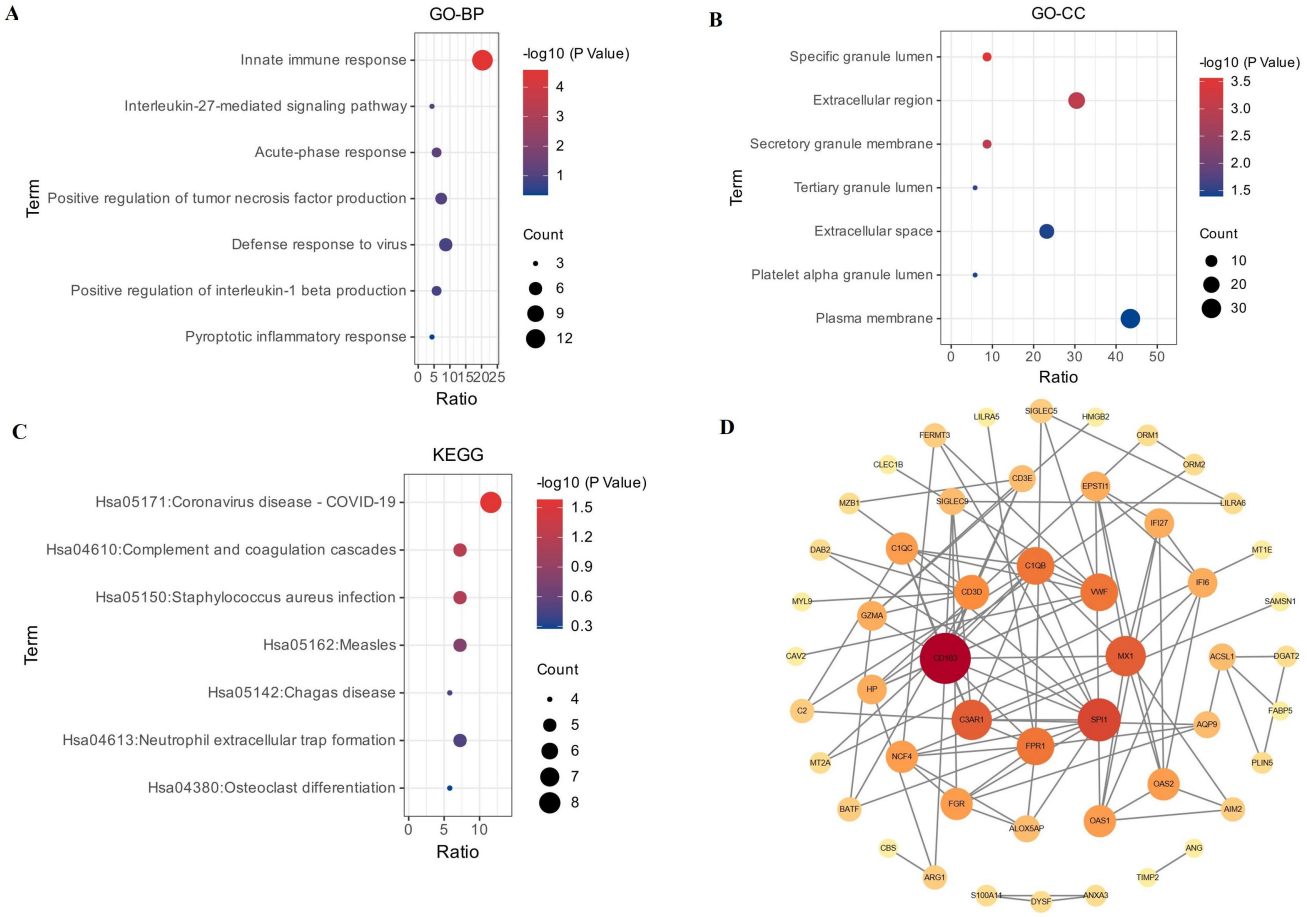
**(A–C)** Bubble plot of GO enrichment analysis and KEGG pathways enrichment analysis. *X-*axis in the figure indicates the ratio of the enriched differential gene to the background gene of the pathway. *Y-*axis shows the names of the different terms. The size of the circle in the plot represented the number of different term and the color changes from blue to red indicated the *p-*value. **(A)** Bubble plot of GO biological processes (GO-BP). **(B)** Bubble plot of GO cellular component (GO-CC). **(C)** Bubble plot of KEGG pathway enrichment (red for a higher degree, pale yellow for a lower degree), and the top eight hub genes were. **(D)** PPI network plot of 70 common genes, consisting of 69 nodes and 99 edges, with colors reflecting the score rank calculated, identified by Cytohubba. CD163 has the highest degree (degree = 13).

For these three datasets, Venn diagrams were used to display 70 common DEGs (Figure 2D), which were selected for further GO enrichment and KEGG pathway analyses. According to GO biological processes (GO-BP) enrichment analysis, we found that these DEGs were mainly enriched in innate immune response by a bubble plot (Figure 3A). According to the GO cellular component (GO-CC) enrichment analysis, we found that these DEGs were mainly associated with the specific granule lumen by a bubble plot (Figure 3B). According to KEGG enrichment analysis, we found these DEGs were mainly enriched in the Coronavirus disease, COVID-19, and complement and coagulation cascade pathways by the bubble plot (Figure 3C).

### 3.5 PPI network and hub genes

We analyzed 70 common DEGs using the STRING database, and we constructed a network graph of PPI, which included 69 nodes and 99 edges. Furthermore, CytoHubba, a plug-in Cytoscape software, was used to identify the top eight hub genes in PPI networks through the degree method. CD163 has the highest degree (degree = 13) and interactions in the PPI network (Figure 3D).

### 3.6 Diagnostic performance of FVIII, FXII, and their ratio in VTE, pneumonia, and sepsis

The diagnostic performance of FVIII, FXII, and the FVIII/FXII ratio was evaluated and compared across the three disease groups (VTE, pneumonia, and sepsis) using ROC analysis.

**Figure 4:**
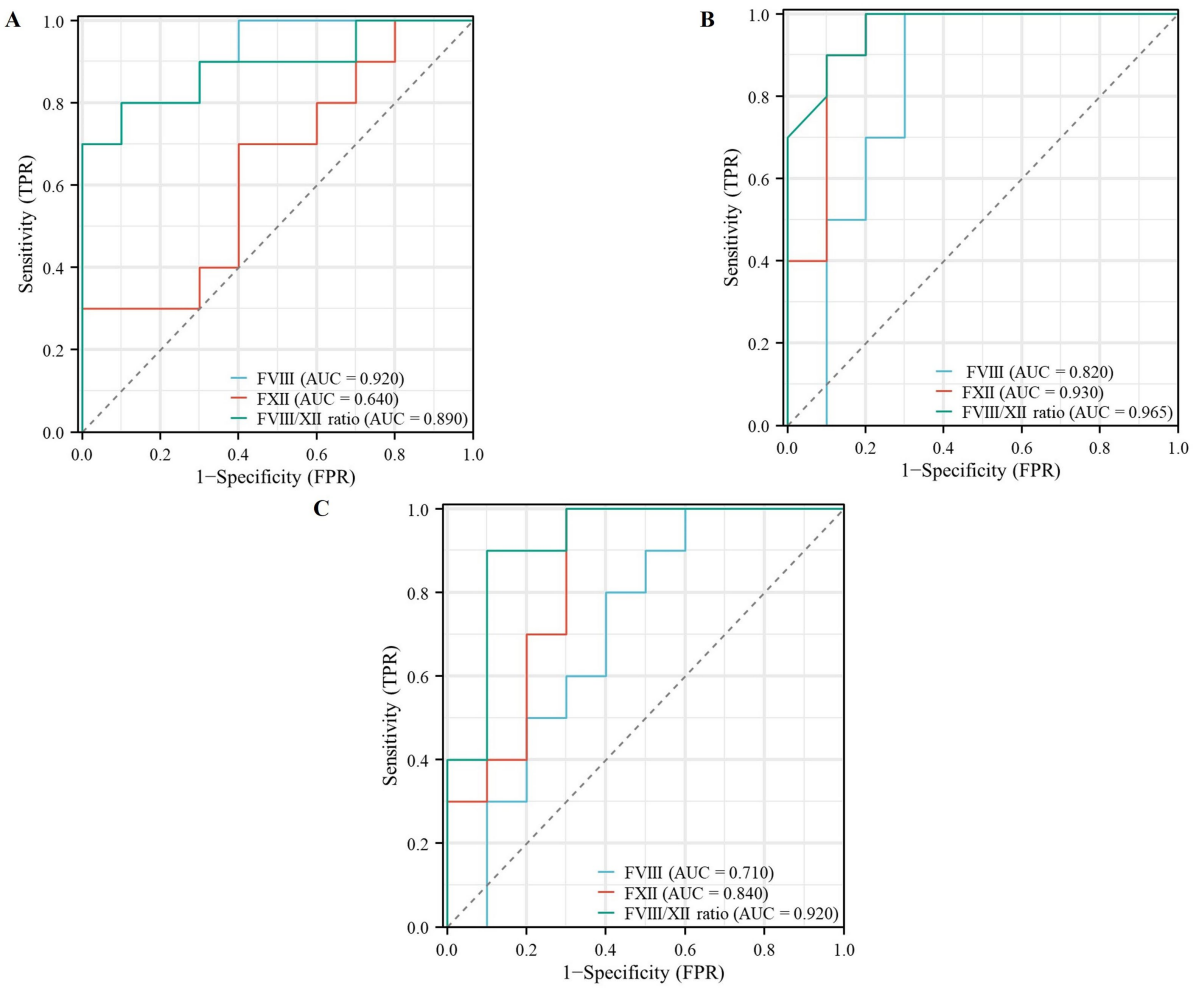
Receiver operating characteristic (ROC) curves evaluating the diagnostic performance of FVIII, FXII, and the FVIII/FXII ratio across the three disease groups (VTE, pneumonia, and sepsis). ROC curves were generated to assess the sensitivity and specificity of each biomarker. Panels **(A,B)** show the corresponding ROC curves for the diagnosis of VTE and pneumonia, respectively, using the same predictors. The FVIII/FXII ratio **(C)** demonstrated significantly superior discriminative ability for sepsis, with an area under the curve (AUC) of 0.920, which was significantly higher than that of FVIII (AUC = 0.710) or FXII (AUC = 0.840) alone.

Based on ROC curve analyses, we found that FVIII yielded a ROC-plot AUC value of 0.920, FXII yielded a ROC-plot AUC value of 0.640, and the FVIII/FXII ratio yielded a ROC-plot AUC value of 0.890 (Figure 4A). The AUC value under the VTE-related ROC curve of FVIII is superior to that of the FXII and the FVIII/FXII ratio.

Based on ROC curve analyses, we found that FVIII yielded a ROC-plot AUC value of 0.820, FXII yielded a ROC-plot AUC value of 0.930, and the FVIII/FXII ratio yielded a ROC-plot AUC value of 0.965 (Figure 4B). The AUC value under the pneumonia-related ROC curve of FXII and the FVIII/FXII ratio is both better than FVIII.

Based on receiver operating characteristic (ROC) curve analyses, the FVIII/FXII ratio demonstrated significantly superior discriminative ability for sepsis, with an area under the curve (AUC) of 0.920 (95% CI: 0.865–0.975, *p* < 0.001). This AUC was significantly higher than those of FVIII (AUC = 0.710, 95% CI: 0.598–0.822) and FXII (AUC = 0.840, 95% CI: 0.750–0.930) alone (Figure 4C).

## 4 Discussion

In the present study, retrospective screening of 1,245 clinical samples tested for intrinsic coagulation factors led to the discovery of a distinct subpopulation characterized by the co-occurrence of elevated FVIII (>150%) and reduced FXII (<50%) activity. This dual abnormality was most frequently associated with three specific conditions: cirrhosis, pneumonia, and sepsis. The selection of these thresholds was guided by both empirical observation in our exploratory cohort and established clinical benchmarks: an FVIII level >150% is associated with a significantly increased risk of venous thrombosis (14), while FXII activity <50% is the widely accepted diagnostic criterion for FXII deficiency (15). Following this phenotypic discovery, we employed bioinformatics approaches to identify common biomarkers and pathways across these three diseases, aiming to elucidate the functional interplay and potential new clinical implications of FVIII and FXII in systemic inflammation and coagulation.

In this study, the publicly available GEO (see text footnote 3) datasets of patients suffering from cirrhosis (GSE54238), pneumonia (GSE171110), and sepsis (GSE137342) were extracted. A total of 70 common DEGs across the three GEO datasets were analyzed. We also investigated 70 common DEGs for GO and KEGG pathways enrichment analyses. GO analysis indicated that these common DEGs were significantly enriched in innate immune response (BP) and specific granule lumen (CC). Host immunity is divided into innate and adaptive immune responses (16). Remote organ inflammation may also result from cellular mediators, both as part of the innate and adaptive immune response (17). Some studies underline the complexity and the importance of the neutrophil responses in host defense in the acute inflammatory response and in subsequent resolution or initiation of innate and specific immune responses (18). Specific granule lumen is primarily found in mature neutrophil cells, and most are released into the extracellular fluid. Neutrophils are generally associated with an inflammatory response (19). Neutrophils are part of the innate immune system that forms the first line of defense against foreign pathogens and foreign bodies. Furthermore, they play an important role in the elimination of cellular “debris” in sterile inflammation. During bacterial infection, neutrophils use various strategies to clear or contain invading pathogens (20). KEGG-based enrichment analysis of the Coronavirus disease (COVID-19) and complement and coagulation cascades suggested activation of the coagulation pathway as well. There is literature evidence that thrombotic complications are a major cause of morbidity and mortality in patients with COVID-19, highlighting the roles of coagulopathy in COVID-19 pathogenesis (21). There is also literature evidence that immune cells can induce local thrombosis as an intravascular defense strategy to pathogen infection. Innate immune cells activate procoagulant pathways to compartmentalize, retain, and kill pathogens (22). The coagulation and complement cascades are designed to act locally—coagulation at sites of injury and complement at sites of infection (23). The common conclusion from the numerous studies linking complement and coagulation is that complement activation leads to initiation or enhancement of coagulation (24). The interaction of the complement and coagulation systems is complex; however, activation of complement is well-established as pro-thrombotic (25). Thus, GO and KEGG functional enrichment analysis revealed the enrichment of pathways associated with inflammation and thrombosis.

STRING database online was used to perform PPI network analysis of the70 common DEGs. In the PPI analysis, the connections between nodes were visualized to identify the interactions between the 70 common DEGs, which included 69 nodes and 99 edges. CytoHubba, a plug-in Cytoscape software, was used to identify the top eight hub genes in PPI networks through the degree method. Meanwhile, CD163 exhibited the highest degree of interaction (degree = 13) and was recognized as a key node in the PPI network. CD163 is a member of the scavenger receptor cysteine-rich superfamily and is exclusively expressed in monocytes and macrophages (26). It functions as an acute phase-regulated receptor involved in the clearance and endocytosis of hemoglobin/haptoglobin complexes by macrophages, and may thereby protect tissues from free hemoglobin-mediated oxidative damage. This protein may also function as an innate immune sensor for bacteria and an inducer of local inflammation. Some studies have shown that CD163 expression is associated with the inhibition of inflammation in both chronic and acute settings (27). Therefore, CD163 is an anti-inflammatory marker. However, inflammation also has a direct impact on the coagulation cascade; thrombogenic factors upregulated by inflammation lead to hypercoagulation and systemic inflammation (28). Given the central role of CD163 in modulating inflammatory processes and its high connectivity in the PPI network identified in our analysis, we propose that CD163 is not merely an anti-inflammatory marker but also a key molecular link between inflammation and coagulation dysregulation. This supports our conclusion that CD163 is critically associated with the pathophysiology of sepsis, where uncontrolled inflammation and coagulopathy are hallmark features.

One of the conclusions from the study above is that the number of specimens with FVIII > 150% (high) and FXII < 50% (low) is the largest. As a consequence, we will focus on FVIII and FXII in the following studies. Previous studies have shown that increased plasma FVIII levels are associated with venous thrombosis (29). FVIII directly promotes thrombosis mainly via increased thrombin generation, resulting in accelerated platelet activation and increased thrombus stability (30). FXII is the initiating protease of the procoagulant and proinflammatory contact system, and it drives both the intrinsic pathway of coagulation and the kallikrein–kinin system (31). FXIIa, in turn, triggers thrombosis via the FXI-mediated intrinsic coagulation pathway. FXIIa also activates PK that produces BK from HK, which drives inflammation (32). Previous studies reported decreased plasma levels of zymogen FXII, plasma prekallikrein (PK), and high-molecular-weight kininogen in patients with sickle cell disease (SCD), suggesting that these proteins may be consumed due to chronic activation (4). Inflammatory mediators can induce endothelial injuries and further activate the clotting system (33). When human blood is exposed to bacterial surfaces, which can bind the zymogen FXII, this factor autoactivates into an enzyme, leading to subsequent thrombin formation *in vitro* (34). Therefore, the reduction of FXII may be associated with persistent infection *in vivo*.

To independently and quantitatively assess the diagnostic potential of this coagulation imbalance, we conducted a formal validation study. A separate validation cohort was established, comprising three distinct patient groups—venous thromboembolism (VTE, *n* = 20), pneumonia (*n* = 20), and sepsis (*n* = 20)—alongside a matched healthy control group (*n* = 20). This cohort was explicitly independent from the samples used in the initial phenotypic screening. Receiver-operating-characteristic (ROC) curve analysis was performed to evaluate the discriminatory power of FVIII, FXII, and their ratio (FVIII/FXII). Our findings indicated that the AUC value under the VTE-related ROC curve of FVIII (0.920) is superior to that of FXII (0.640) and the FVIII/FXII ratio (0.890). The AUC value under the pneumonia-related ROC curve of FXII (0.930) and the FVIII/FXII ratio (0.965) is superior to FVIII (0.820). The AUC value of the FVIII/FXII ratio in diagnosing sepsis reached 0.92, which was significantly higher than that of FVIII (0.71) and FXII (0.84) alone. The results demonstrated that the FVIII/FXII ratio exhibited superior diagnostic accuracy for sepsis, as evidenced by the largest area under the curve (AUC). This supports the hypothesis that the ratio, integrating both prothrombotic (high FVIII) and inflammatory (low FXII) dimensions, provides a more robust biomarker for sepsis than either factor alone. This superior performance may be attributed to the fact that the ratio more comprehensively reflects the coordinated dysregulation of coagulation and inflammatory pathways in sepsis. While FVIII, as an acute-phase protein, increases with systemic inflammation, and FXII often decreases due to consumption in continuous activation of the contact system, the FVIII/FXII ratio amplifies these opposing trends, thereby enhancing the sensitivity and specificity in distinguishing septic patients from non-septic individuals. Hence, we thus inferred that FVIII is most related to thrombus and is good in predicting thrombus, and FXII is most related to inflammation and is good in predicting inflammation. However, in sepsis patients, the FVIII/FXII ratio is associated with both thrombus and inflammation. Since FVIII is associated with thrombus, and FXII is related to inflammation, both have been demonstrated in previous studies, but the FVIII/FXII ratio is the uniqueness of the question, which has never been investigated in other institutions. Ample evidence has shown that severe sepsis is accompanied by both activation of a strong proinflammatory response and increased coagulation activation. Severe inflammation leads to the activation of coagulation, and coagulation also affects the progression of inflammation (35). In sepsis patients, inflammation and coagulation interact with each other. This interaction is evidenced by concurrent increases in FVIII and decreases in FXII. The severity of the disease is correlated with the magnitude of this ratio. Consequently, we propose that the FVIII/FXII ratio holds potential as an adjunctive biomarker to aid in the early detection of sepsis.

Sepsis resulting from a generalized inflammatory and procoagulant response to an infection is associated with a high risk of mortality (36). Early detection is critical, as it enables timely intervention and can potentially improve outcomes. Beyond the standalone performance of the FVIII/FXII ratio, our study provides preliminary but mechanistically grounded evidence for its collaborative potential with CD163. While the FVIII/FXII ratio quantifies a plasma-based imbalance between prothrombotic and proinflammatory drivers within the coagulation cascade, CD163 serves as a cellular marker, reflecting the state of monocyte/macrophage activation, a key orchestrator of the systemic inflammatory response in sepsis. The observed correlation between elevated CD163 levels and an increased FVIII/FXII ratio in our clinical samples suggests that these two markers capture distinct yet interconnected facets of the septic “immunothrombotic” state. This implies that their combined use could offer a more holistic assessment: the FVIII/FXII ratio indicating the severity of the coagulation-inflammation interplay, and CD163 signaling the intensity of the underlying immune cell activation. Future studies with larger cohorts are warranted to formally test a combined panel (e.g., a logistic regression model incorporating both markers) and to determine if it yields superior diagnostic or prognostic accuracy compared to either marker alone, thereby fully realizing their collaborative diagnostic value. In this context, our findings regarding CD163 and the FVIII/FXII ratio hold significant promise for reducing sepsis-related mortality. Critically, our analysis of clinical samples revealed a detectable correlation between elevated CD163 expression levels and an increased FVIII/FXII ratio, suggesting a potential *in vivo* link between monocyte/macrophage activation (as indicated by CD163) and the dysregulation of the coagulation cascade in sepsis. Although the study’s sample size was limited and the design was a retrospective, single-center analysis, the strength and consistency of this observed association—even within this exploratory cohort—underscore its potential biological and clinical relevance. Notably, our model demonstrated high discriminatory power not in a simplified case–control setting, but within a complex multi-disease framework that more accurately reflects real-world clinical scenarios. This enhances the practical validity of our findings. The correlation identified in patient samples between CD163 and the FVIII/FXII ratio provides a crucial foundational insight, solidifying the rationale for further large-scale, multi-center prospective studies to validate their combined utility as biomarkers for sepsis diagnosis and risk stratification.

## 5 Limitations

This study has several limitations. First, the lack of experimental validation, such as RT-PCR or immunohistochemistry on clinical samples. This prevents us from conclusively demonstrating the direct relevance of CD163 to the pathophysiology of sepsis. Future studies are imperative to independently validate the expression and functional role of CD163 in sepsis using well-characterized clinical cohorts, which will be crucial for translating these bioinformatics findings into a deeper pathophysiological understanding. Second, the sample size of this study was limited, particularly within the specific disease subgroups (e.g., sepsis *n* = 20 in ROC analysis), which increases the risk of model overfitting and limits the generalizability of the subgroup-specific findings. Despite these limitations, which we have explicitly acknowledged to ensure transparency, our study provides essential foundational evidence and lays the groundwork for future validation.

## Data Availability

Publicly available datasets were analyzed in this study. This data can be found at: GEO (https://www.ncbi.nlm.nih.gov/geo/), accession numbers: GSE54238, GSE171110, and GSE137342.

## Footnotes

1
https://www.xiantaozi.com/products

2
https://hiplot.com.cn

3
https://www.ncbi.nlm.nih.gov/geo/

4
https://www.ncbi.nlm.nih.gov/geo/geo2r/

5 https://www.xiantaozi.com, accessed on 15 February 2025.

6 https://string-db.org/, accessed on 16 February 2025.

## References

[1] Hoshino K Kitamura T Nakamura Y Irie Y Matsumoto N Kawano Y . Usefulness of plasminogen activator inhibitor-1 as a predictive marker of mortality in sepsis. J Intensive Care. (2017) 5:42. doi: 10.1186/s40560-017-0238-8, 28702197 PMC5504563

[2] Singer M Deutschman CS Seymour CW Shankar-Hari M Annane D Bauer M . The third international consensus definitions for sepsis and septic shock (Sepsis-3). JAMA. (2016) 315:801–10. doi: 10.1001/jama.2016.0287, 26903338 PMC4968574

[3] Chen S Ding R Hu Z Yin X Xiao F Zhang W . MicroRNA-34a inhibition alleviates lung injury in cecal ligation and puncture induced septic mice. Front Immunol. (2020) 11:1829. doi: 10.3389/fimmu.2020.01829, 32903604 PMC7438583

[4] Giza DE Fuentes-Mattei E Bullock MD Tudor S Goblirsch MJ Fabbri M . Cellular and viral microRNAs in sepsis: mechanisms of action and clinical applications. Cell Death Differ. (2016) 23:1906–18. doi: 10.1038/cdd.2016.94, 27740627 PMC5136497

[5] Wada Y Otu H Wu S Abid MR Okada H Libermann T . Preconditioning of primary human endothelial cells with inflammatory mediators alters the "set point" of the cell. FASEB J. (2005) 19:1914–6. doi: 10.1096/fj.05-4037fje, 16172186 PMC5378497

[6] Burzynski LC Humphry M Pyrillou K Wiggins KA Chan JNE Figg N . The coagulation and immune systems are directly linked through the activation of interleukin-1celll death D. Immunity. (2019) 50:e6. doi: 10.1016/j.immuni.2019.03.003, 30926232 PMC6476404

[7] Kager LM Wiersinga WJ Roelofs JJ de Boer OJ Weiler H van 't Veer C . A thrombomodulin mutation that impairs active protein C generation is detrimental in severe pneumonia-derived gram-negative sepsis (melioidosis). PLoS Negl Trop Dis. (2014) 8:e2819. doi: 10.1371/journal.pntd.0002819, 24762740 PMC3998929

[8] Jankowska KI McGill J Pezeshkpoor B Oldenburg J Sauna ZE Atreya CD. Further evidence that MicroRNAs can play a role in hemophilia a disease manifestation: F8 gene downregulation by miR-19b-3p and miR-186-5p. Front Cell Dev Biol. (2020) 8:669. doi: 10.3389/fcell.2020.00669, 32850803 PMC7406646

[9] Sparkenbaugh EM Henderson MW Miller-Awe M Abrams C Ilich A Trebak F . Factor XII contributes to thrombotic complications and vaso-occlusion in sickle cell disease. Blood. (2023) 141:1871–83. doi: 10.1182/blood.2022017074, 36706361 PMC10122107

[10] García-Laorden MI Stroo I Blok DC Florquin S Medema JP de Vos AF . Granzymes A and B regulate the local inflammatory response during *Klebsiella pneumoniae* pneumonia. J Innate Immun. (2016) 8:258–68. doi: 10.1159/000443401, 26894590 PMC6738821

[11] Bruns T Peter J Hagel S Herrmann A Stallmach A. The augmented neutrophil respiratory burst in response to *Escherichia coli* is reduced in liver cirrhosis during infection. Clin Exp Immunol. (2011) 164:346–56. doi: 10.1111/j.1365-2249.2011.04373.x, 21413941 PMC3087930

[12] Li J Miao B Wang S Dong W Xu H Si C . Hiplot: a comprehensive and easy-to-use web service for boosting publication-ready biomedical data visualization. Brief Bioinform. (2022) 23:bbac261. doi: 10.1093/bib/bbac261, 35788820

[13] Tang Y Li M Wang J Pan Y Wu FX. CytoNCA: a cytoscape plugin for centrality analysis and evaluation of protein interaction networks. Biosystems. (2015) 127:67–72. doi: 10.1016/j.biosystems.2014.11.005S, 25451770

[14] Kyrle PA Minar E Hirschl M Bialonczyk C Stain M Schneider B . High plasma levels of factor VIII and the risk of recurrent venous thromboembolism. N Engl J Med. (2000) 343:457–62. doi: 10.1056/nejm200008173430702 10950667

[15] Peyvandi F Palla R Menegatti M Siboni SM Halimeh S Faeser B . Coagulation factor activity and clinical bleeding severity in rare bleeding disorders: results from the European network of rare bleeding disorders. J Thromb Haemost. (2012) 10:615–21. doi: 10.1111/j.1538-7836.2012.04653.x, 22321862

[16] Zarrin AA Bao K Lupardus P Vucic D. Kinase inhibition in autoimmunity and inflammation. Nat Rev Drug Discov. (2021) 20:39–63. doi: 10.1038/s41573-020-0082-8, 33077936 PMC7569567

[17] Ruparelia N Digby JE Jefferson A Medway DJ Neubauer S Lygate CA . Myocardial infarction causes inflammation and leukocyte recruitment at remote sites in the myocardium and in the renal glomerulus. Inflamm Res. (2013) 62:515–25. doi: 10.1007/s00011-013-0605-4 23471223 PMC3625409

[18] Gomez JC Yamada M Martin JR Dang H Brickey WJ Bergmeier W . Mechanisms of interferon-on causes inflammation and leukocyte recruitment at remote sites in the myocardium and. Am J Respir Cell Mol Biol. (2015) 52:349–64. doi: 10.1165/rcmb.2013-0316oc 25100610 PMC4370257

[19] Mukherjee S Laiakis EC Fornace AJ Jr Amundson SA. Impact of inflammatory signaling on radiation biodosimetry: mouse model of inflammatory bowel disease. BMC Genomics. (2019) 20:329. doi: 10.1186/s12864-019-5689-y, 31046668 PMC6498469

[20] Saha B Tornai D Kodys K Adejumo A Lowe P McClain C . Biomarkers of macrophage activation and immune danger signals predict clinical outcomes in alcoholic hepatitis. Hepatology. (2019) 70:1134–49. doi: 10.1002/hep.30617, 30891779 PMC6752989

[21] Gu SX Tyagi T Jain K Gu VW Lee SH Hwa JM . Thrombocytopathy and endotheliopathy: crucial contributors to COVID-19 thromboinflammation. Nat Rev Cardiol. (2021) 18:194–209. doi: 10.1038/s41569-020-00469-1, 33214651 PMC7675396

[22] Riegger J Byrne RA Joner M Chandraratne S Gershlick AH Ten Berg JM . Histopathological evaluation of thrombus in patients presenting with stent thrombosis. A multicenter European study: a report of the prevention of late stent thrombosis by an interdisciplinary global European effort consortium. Eur Heart J. (2016) 37:1538–49. doi: 10.1093/eurheartj/ehv419, 26761950 PMC4872283

[23] Gulla KC Gupta K Krarup A Gal P Schwaeble WJ Sim RB . Activation of mannan-binding lectin-associated serine proteases leads to generation of a fibrin clot. Immunology. (2010) 129:482–95. doi: 10.1111/j.1365-2567.2009.03200.x 20002787 PMC2842495

[24] Jokiranta TS. HUS and atypical HUS. Blood. (2017) 129:2847–56. doi: 10.1182/blood-2016-11-709865, 28416508 PMC5445567

[25] Prendecki M Clarke C Medjeral-Thomas N McAdoo SP Sandhu E Peters JE . Temporal changes in complement activation in haemodialysis patients with COVID-19 as a predictor of disease progression. Clin Kidney J. (2020) 13:889–96. doi: 10.1093/ckj/sfaa192, 33123364 PMC7577776

[26] Klimczak-Tomaniak D Bouwens E Schuurman AS Akkerhuis KM Constantinescu A Brugts J . Temporal patterns of macrophage- and neutrophil-related markers are associated with clinical outcome in heart failure patients. ESC Heart Fail. (2020) 7:1190–200. doi: 10.1002/ehf2.12678, 32196993 PMC7261550

[27] Ye H Wang LY Zhao J Wang K. Increased CD163 expression is associated with acute-on-chronic hepatitis B liver failure. World J Gastroenterol. (2013) 19:2818–25. doi: 10.3748/wjg.v19.i18.2818, 23687420 PMC3653157

[28] Ogdie A Kay McGill N Shin DB Takeshita J Jon Love T Noe MH . Risk of venous thromboembolism in patients with psoriatic arthritis, psoriasis and rheumatoid arthritis: a general population-based cohort study. Eur Heart J. (2018) 39:3608–14. doi: 10.1093/eurheartj/ehx145, 28444172 PMC6186275

[29] Poothong J Pottekat A Siirin M Campos AR Paton AW Paton JC . Factor VIII exhibits chaperone-dependent and glucose-regulated reversible amyloid formation in the endoplasmic reticulum. Blood. (2020) 135:1899–911. doi: 10.1182/blood.2019002867, 32128578 PMC7243144

[30] Machlus KR Lin FC Wolberg AS. Procoagulant activity induced by vascular injury determines contribution of elevated factor VIII to thrombosis and thrombus stability in mice. Blood. (2011) 118:3960–8. doi: 10.1182/blood-2011-06-362814, 21828144 PMC3193271

[31] Wilbs J Kong XD Middendorp SJ Prince R Cooke A Demarest CT . Cyclic peptide FXII inhibitor provides safe anticoagulation in a thrombosis model and in artificial lungs. Nat Commun. (2020) 11:3890. doi: 10.1038/s41467-020-17648-w, 32753636 PMC7403315

[32] Maas C Renné T. Coagulation factor XII in thrombosis and inflammation. Blood. (2018) 131:1903–9. doi: 10.1182/blood-2017-04-569111 29483100

[33] Han C Han L Huang P Chen Y Wang Y Xue F. Syncytiotrophoblast-derived extracellular vesicles in pathophysiology of preeclampsia. Front Physiol. (2019) 10:1236. doi: 10.3389/fphys.2019.01236, 31632289 PMC6779799

[34] Siemens N Oehmcke-Hecht S Hoßmann J Skorka SB Nijhuis RHT Ruppen C . Prothrombotic and proinflammatory activities of the ia a thrombosis model and in artificial. J Innate Immun. (2020) 12:291–303. doi: 10.1159/000504002, 31743913 PMC7383282

[35] Liu Y Xiang D Zhang H Yao H Wang Y. Hypoxia-inducible factor-1: a potential target to treat acute lung injury. Oxidative Med Cell Longev. (2020) 2020:8871476. doi: 10.1155/2020/8871476, 33282113 PMC7685819

[36] Allingstrup M Wetterslev J Ravn FB Møller AM Afshari A. Antithrombin III for critically ill patients: a systematic review with meta-analysis and trial sequential analysis. Intensive Care Med. (2016) 42:505–20. doi: 10.1007/s00134-016-4225-7, 26862016 PMC7095103

